# Clinical cure rate of inactive HBsAg carriers with HBsAg <200 IU/ml treated with pegylated interferon

**DOI:** 10.3389/fimmu.2022.1091786

**Published:** 2022-12-23

**Authors:** Hong Li, Shan Liang, Lili Liu, Daqiong Zhou, Yali Liu, Yang Zhang, Xinyue Chen, Jing Zhang, Zhenhuan Cao

**Affiliations:** ^1^ The Third Unit, Department of Hepatology, Beijing Youan Hospital, Capital Medical University, Beijing, China; ^2^ Beijing Institute of Hepatology, Beijing Youan Hospital, Capital Medical University, Beijing, China; ^3^ The First Unit, Department of Hepatology, Beijing Youan Hospital, Capital Medical University, Beijing, China

**Keywords:** inactive HBsAg carriers, HBsAg clearance, pegylated interferon, hepatic steatosis, clinical cure

## Abstract

**Purpose:**

HBsAg clearance represents clinical cure for patients with hepatitis B, but remains difficult to obtain for most HBV-infected patients. Recent studies have shown that inactive HBsAg carriers treated with pegylated interferon can achieve higher clinical cure rates, which may imply that the lower the baseline HBsAg quantification, the higher HBsAg clearance rate. Therefore, this study further investigated the HBsAg clearance rate in inactive HBsAg carriers with low level of HBsAg (<200 IU/ml) treated with pegylated interferon.

**Methods:**

This is a prospective cohort study. Inactive HBsAg carriers with HBsAg<200 IU/ml were divided into treatment and control groups. Pegylated interferon was administered to the patients in therapeutic group for 96 weeks. The patients in control group underwent 96 weeks of observation without any anti-viral treatment. All patients were tested for HBsAg, anti-HBs, HBV DNA, liver function, blood count, thyroid function, thyroid antibodies and autoantibodies at baseline, week 12, 24, 36, 48, 60, 72, 84 and 96. Controlled attenuation parameter (CAP) and liver stiffness measure (LSM) were evaluated at baseline and week 96. Patients were classified into no steatosis, mild steatosis, moderate steatosis and severe steatosis according to the value of CAP.

**Results:**

A total of 174 inactive HBsAg carriers with HBsAg<200IU/ml were enrolled, including 84 in the treatment group and 90 in the control group. In the treatment group, HBsAg clearance rate was 30.77% (24/78) at week 48, and increased to 57.69% (45/78) at week 96. HBsAg clearance occurred in 2 patients with a clearance rate of 2.27% (2/88) in control group, The HBsAg clearance rate of the treatment group was significantly higher than that of the control group (P<0.001). HBsAg clearance was significantly higher in patients with moderate steatosis than in those without steatosis (74.07% vs. 48.15%, p=0.008) at week 96.

**Conclusion:**

High HBsAg clearance rate could be obtained for inactive HBsAg carriers with HBsAg< 200 IU/ml treated with peginterferons. Inactive HBsAg carriers with moderate hepatic steatosis are more sensitive for the treatment.

## Introduction

Approximately 250 million people worldwide are chronically infected with HBV estimated by The World Health Organization (1) Inactive HBsAg Carriers (IHCs) characterized by HBsAg <1000 IU/ml, negative HBeAg, HBV DNA <2000 IU/ml, normal ALT (2) account for about 36% of the whole population infected with hepatitis B virus (HBV) (3). IHCs can convert to HBeAg-positive chronic hepatitis B (CHB), or to HBeAg-negative CHB due to viral mutation and immune escape (4, 5). A long-term follow-up on IHCs found that the cumulative recurrent rates of hepatitis were 10.2%, 17.4%, 19.3%, 20.2% and 20.2% at five, ten, fifteen, twenty, and twenty-five years, respectively (6). Compared to healthy individuals (HBsAg-negative), IHCs have 4.6-fold and 2.1-fold higher mortality associated with liver cancer and liver disease respectively (7, 8). Recent studies have confirmed that some IHCs could achieve HBsAg clearance or serconversion after pegylated interferon (PEG-IFN) treatment, and there is a correlation between baseline HBsAg quantification and HBsAg clearance, probably the lower level of baseline HBsAg, the higher rate of HBsAg clearance (9). This study further explored the clinical cure rate and safety of IHCs with HBsAg <200 IU/ml treated with PEG-IFN for 96 weeks.

## Patients and methods

### Patients

All IHCs met the criteria defined in the prevention and treatment guidelines for chronic hepatitis B (2019 edition). (i) the history of HBsAg positive was longer than 6 months and the level of HBsAg lower than 200 IU/mL, HBeAg negative, anti-HBe negative/positive; (ii) HBV DNA < 20 IU/mL, Alanine aminotransferase (ALT) normal (male < 50 IU/L, female < 40 IU/L); (iii) white blood cell count > 4×10^9^/L, platelet count >150×10^9^/L; (iv) Total bilirubin (TBil) <34 μmol/L, albumin > 40 g/L. Exclusion criteria: (i) history of autoimmune diseases; (ii) human immunodeficiency virus (HIV) or hepatitis C virus (HCV) or hepatitis E virus(HEV) coinfected; (iii) pregnant, lactating women and those who are preparing to be pregnant; (iv) liver cirrhosis or liver cancer; (v) history of severe heart disease; (vi) history of mental illness or psychiatric disorders. (vii) uncontrolled epilepsy. (viii) alcohol or drug abuser. (ix) uncontrolled diabetes mellitus, hypertension, thyroid disease, retinopathy; (x) contraindication to interferon.

### Ethics approval

All patients signed an informed consent form before enrollment. The protocol and the consent form for the study were approved by the research ethics committee of Beijing Youan Hospital, Capital Medical University, China ([2017]24).

### Treatment and efficacy

This is a prospective cohort study. Patients in treatment group were given PEG-IFN180ug subcutaneously once a week for 96 weeks. Patients in the control group were observed for 96 weeks without anti-viral treatment. Patients in the treatment group were divided into HBsAg clearance group (Responders, R group) and non-HBsAg clearance group (Non-responders, NR group) according to whether HBsAg clearance was achieved within 96 weeks. Patients were divided into four groups according to controlled attenuation parameter (CAP)value at baseline: no hepatic steatosis (CAP < 238dB/m), mild steatosis (238dB/m < CAP < 250dB/m), moderate steatosis (250dB/m ≤ CAP < 292dB/m) and severe steatosis (CAP ≥ 292dB/m).

### Laboratory tests

All patients were tested with HBsAg, anti-HBs, HBV DNA, liver function, blood count, thyroid function, thyroid antibodies and autoantibodies at baseline, and at the time point of week 12, 24, 36, 48, 60, 72, 84 and 96. CAP and liver stiffness measure (LSM) were evaluated at baseline and week 96. HBsAg was quantified with HBsAg quantification kit (Roche, Mannheim, Germany), the lower limit of detection of HBsAg was 0.05 IU/mL. HBV DNA was analyzed by cobas^®^ AmpliPrep/cobas^®^ Taqman automated nucleic acid isolation and purification and PCR system (Roche Diagnostics GmbH, Mannheim, Germany), the lower limit of detection of HBV DNA was 20 IU/ml. ALT was measured by OLYMPUS-AU5400 (Japan), normal value < 50 IU/L for men and < 40 IU/L for women. Thyroid antibodies were detected using RocheCobase801, with normal reference values: anti-thyroglobulin antibody (TGAb) < 115IU/ml, anti-thyroid peroxidase antibody (TPOAb) < 34IU/ml, thyrotropin receptor antibody (TRAb) < 1.75IU/ml. Thyroid function was measured by Architecti2000 (Abbott, Mannheim, USA) with normal reference ranges: FT3 ranged from 3.6 to 6.5pmol/L, TT3 ranged from 0.92-2.79 nmol/L, FT4 ranged from 11.5 to 22.7pmol/L, TT3 ranged from 58.1-140.6 nmol/L, and TSH ranged from 0.55-4.78 mIU/L. CAP and LSM were measured by transient elastic scanner (Fibroscan‐502, M probe, frequency 3.5M Hz, Echosens, France).

### Statistical analysis

Statistical software package SPSS (SPSS Inc., USA) 25.0 software was used for statistical analyses. Normally distributed parameters were expressed as mean ± standard deviation and compared by t-test. (ANOVA for more than 2 groups) Data that were not normally distributed were expressed as median (25th, 75^th^ range) and non-parametric tests were used for comparison between groups. The Kaplan-Meier survival analysis was used to calculate the cumulative HBsAg clearance rate. P-value <0.05 was considered statistically significant. In this study, the subjects who completed 96-week trial after enrolment were analyzed without regard of dropped off or loss of follow-up, i.e. using per-protocol analysis (PP analysis).

## Results

### Therapy outcomes

**Figure 1:**
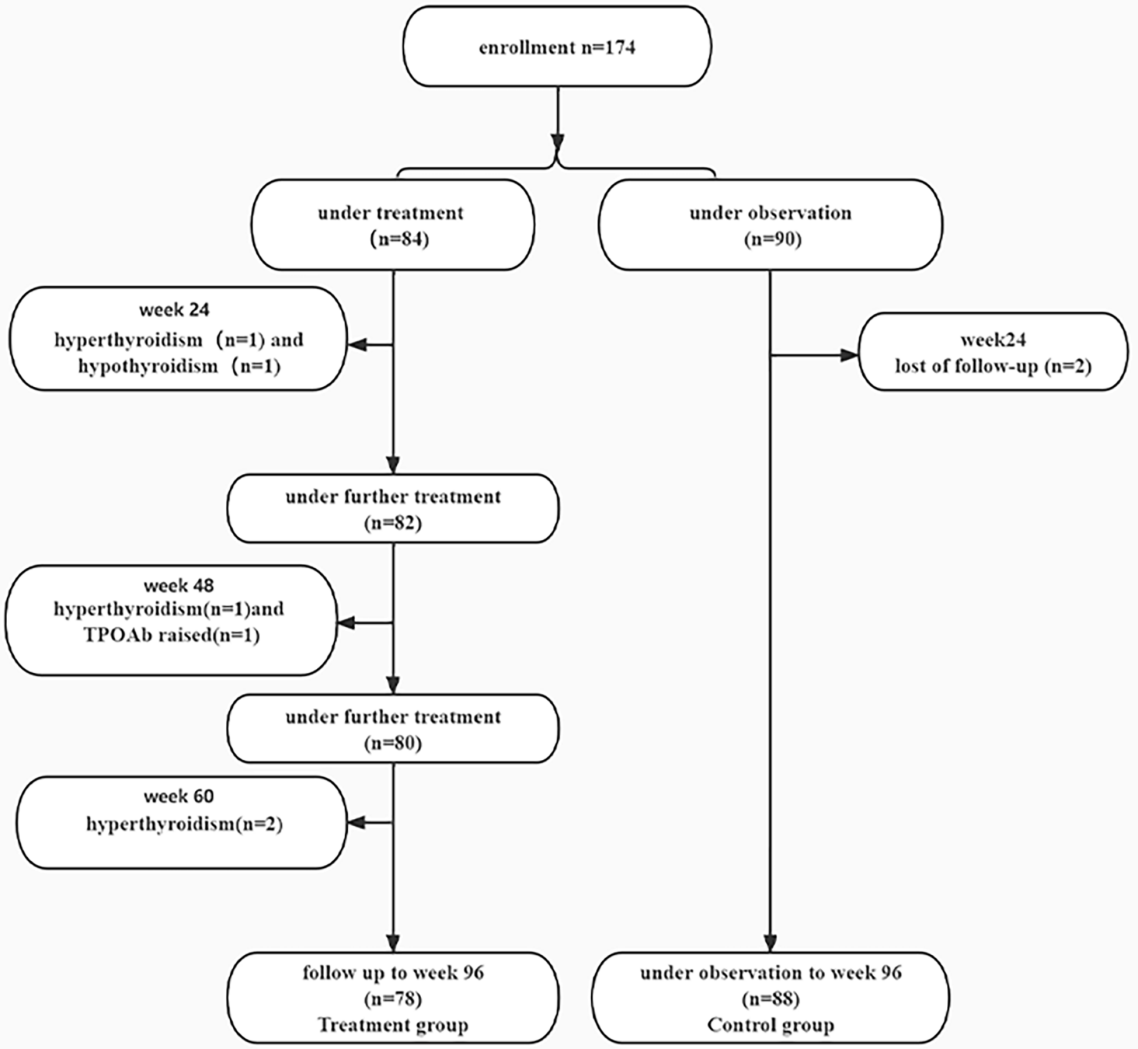
Flow diagram of participants enrolled in this study.

A total of 174 IHCs were enrolled, 84 patients received PEG-IFN, 90 patients did not receive anti-viral treatment. Two patients in the treatment group discontinued PEG-IFN at week 24 due to hyperthyroidism and one patient stopped treatment due to hypothyroidism. At week 48, one patient stopped treatment due to hyperthyroidism and one patient discontinued treatment because of an increase of TPOAb (355.3 IU/ml); One patient stopped PEG-IFN due to hyperthyroidism at week 60. 78 patients completed the 96-week course of treatment. 88 patients in the control group were followed up to week 96 and two subjects were lost follow-up at week 24 (**Figure 1**).

### Baseline characteristics

There were no statistical differences in gender, age and HBsAg quantification between the two groups (**Table 1**).

**Table 1:** Baseline characteristics.

Parameter	Treatment group n=78	Control group n=88	P-value
Gender (M/F)	54.00/24.00	48.00/40.00	0.052
Age (years)	42.03 ± 8.16	44.44 ± 9.25	0.078
ALT (U/L)	26.50 (16.00, 32.00)	22 (16.00, 30.50)	0.129
HBsAg (IU/mL)	70.99 (6.19, 161.90)	55.61 (4.31, 113.60)	0.166
Mode of transmission			
Vertical (%)	46.43% (39.00/78.00)	45.56% (41.00/88.00)	0.908
Others (%)	53.57% (45.00/78.00)	54.44% (49.00/88.00)	

### HBsAg clearance rate in treatment and control group

**Figure 2:**
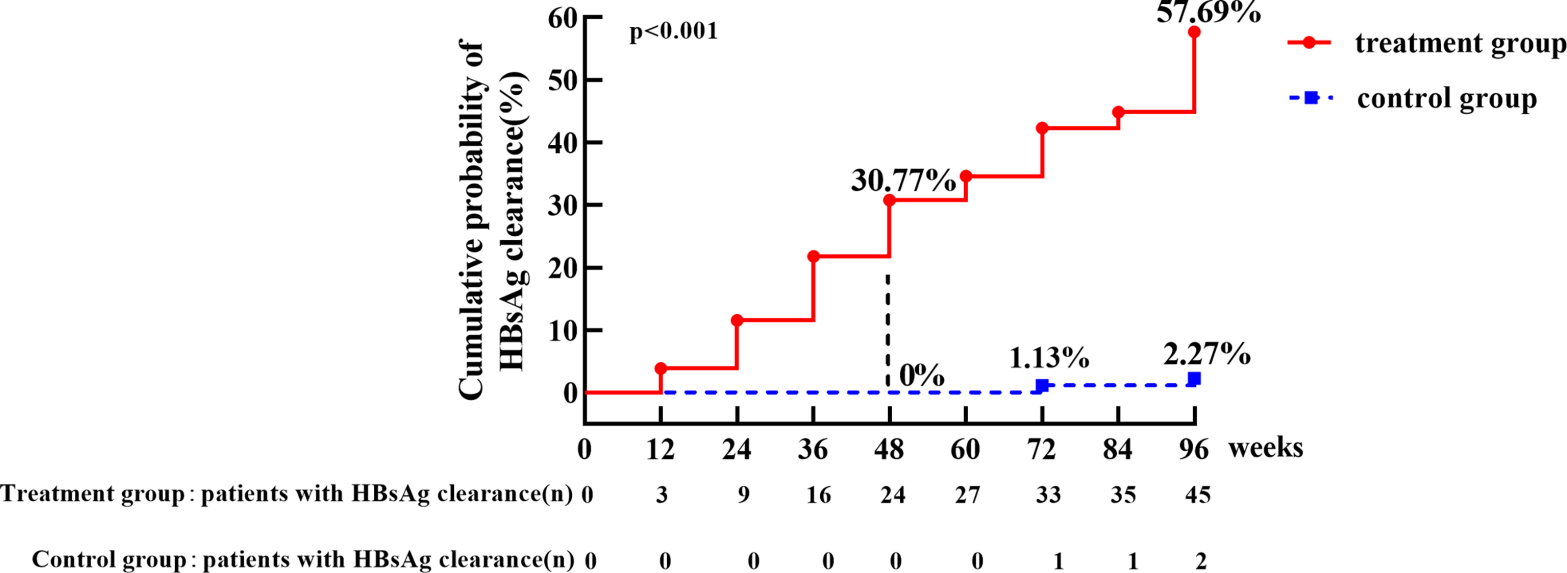
The cumulative incidence of HBsAg clearance in treatment group and control group. The cumulative rate of HBsAg clearance at week 48 was 30.77% in treatment group versus 0% in the control group, respectively. The cumulative incidence of HBsAg clearance was 57.69% in treatment group, which was significantly higher than control group (2.27%) at week 96, p < 0.001.

In the treatment group, the HBsAg clearance rate was 30.77% (24/78) and HBsAg seroconversion rate was 23.08% (18/78) at week 48. HBsAg clearance rate increased to 57.69% (45/78) and HBsAg seroconversion rate was 55.13% (43/78) at week 96. In the control group, spontaneous HBsAg clearance occurred in two patients (one with baseline HBsAg 0.202 IU/mL and HBsAg cleared at week 72, the other with baseline HBsAg 3.67 IU/mL and cleared at week 96, with a clearance rate of 2.27% (2/88) and no patient achieved seroconversion. HBsAg clearance rate was significantly higher in the treatment group than that in the control group (p < 0.001) (**Figure 2**).

### HBsAg level in treatment group

**Figure 3:**
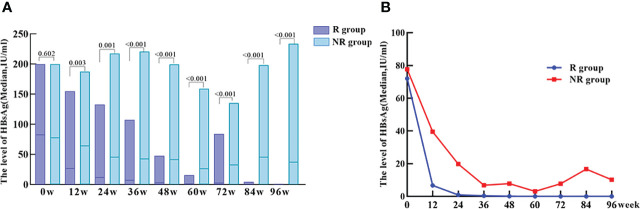
The level of HBsAg at baseline and during treatment of PEG-IFN in R and NR groups. **(A)** No significant difference of HBsAg level between R and NR group at baseline (p = 0.602). The level of HBsAg in R group was significantly lower than NR group during treatment (p = 0.003, p = 0.001, p < 0.001, p < 0.001, p < 0.001, p < 0.001, p < 0.001, P < 0.001) **(B)** HBsAg showed a significant downward trend after PEG-IFN treatment in IHCs, especially in R group.

A total of 45 patients in the treatment group achieved HBsAg clearance and were classified as responder group (R group). 33 patients did not achieve HBsAg clearance and were classified as non-Responder group (NR group). At baseline, there was no statistically significant difference in HBsAg levels between R and NR group (p = 0.602), and HBsAg quantification was significantly lower in R group than that in NR group at 12, 24, 36, 48, 60, 72, 84 and 96 weeks of PEG-IFN treatment (p = 0.003, p = 0.001, p < 0.001, p < 0.001, p<0.001, p < 0.001, p < 0.001, p < 0.001 respectively). In addition, patients in R group had a significantly greater decrease of HBsAg level at each time point during PEG-IFN treatment than those in NR group (p = 0.031, p = 0.019, p = 0.124, p = 0.002, p = 0.059, p = 0.017, p = 0.002, p < 0.001, respectively). (**Table 2**; **Figure 3**).

**Table 2:** Longitudinal changes of HBsAg level during treatment.

Parameter	R group n=45	NR group n=33	P-value
HBsAg level (IU/mL)			
Baseline	72.04 (3.45, 164.75)	77.67 (19.16, 167.95)	0.602
Week12	6.75 (0.77, 36.82)	39.52 (7.99, 126.65)	0.003*
Week24	0.96 (0.13, 5.42)	19.83 (0.70, 71.14)	0.001*
Week36	0.40 (3.45, 1.24)	6.86 (0.76, 76.00)	<0.001*
Week48	0.11 (0.00, 0.66)	7.83 (0.58, 64.09)	<0.001*
Week60	0.03 (0.00, 0.57)	3.13 (0.91, 36.16)	<0.001*
Week72	0.00 (0.00, 0.08)	7.74 (1.26, 58.30)	<0.001*
Week84	0.00 (0.00, 0.06)	16.71 (2.29, 76.25)	<0.001*
Week96	0.00 (0.00, 0.00)	10.18 (4.73, 58.01)	<0.001*
HBsAg decline from baseline (log_10_ IU/ml)			
Week12	0.60 ± 0.13	0.24 ± 0.10	0.031*
Week24	1.12 ± 0.16	0.58 ± 0.14	0.019*
Week36	1.24 ± 0.22	0.79 ± 0.18	0.124
Week48	1.43 ± 0.15	0.73 ± 0.16	0.002*
Week60	1.31 ± 0.18	0.84 ± 0.16	0.059
Week72	1.38 ± 0.17	0.76 ± 0.17	0.017*
Week84	1.41 ± 0.18	0.50 ± 0.23	0.002*
Week96	1.39 ± 0.14	0.56 ± 0.14	<0.001*

R group (Responder group); NR group (Non-Responder group). * p <0.05 .

### ALT level during PEG-IFN treatment in R and NR group

The level of ALT in R group showed significantly higher at week 12 [48.0 (27.0, 72.0)] than baseline [26.0 (15.5, 38.5), p<0.001]. ALT level in NR group also showed significantly higher at week 12 [38.0 (26.5, 64.8)] than baseline [27.0 (18.0, 39.0), p=0.001].

**Figure 4:**
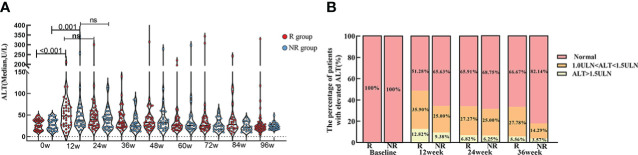
ALT level at baseline and during PEG-IFN treatment in R and NR group. **(A)** ALT level was significant higher both in R and NR group at week 12 than baseline (p < 0.001, p = 0.001). ALT level were not statistically different at each time point between R and NR group. **(B)** The proportion of patients with elevated level of ALT in R group was marginally larger than NR group, although there was no statistically significant difference.

In spite of that the ALT level in R and NR group were not diverse at each time point, the proportion of patients with elevated ALT [1.0-1.5 upper limit of normal (ULN) and ALT > 1.5 (ULN)] was slightly higher in R group than NR group, especially at week 12. (**Table 3**; **Figure 4**).

**Table 3:** ALT level at baseline and during treatment.

Parameter	R group n=45	NR group n=33	P-value
Baselines	26.00 (15.50, 38.50)	27.00 (18.00, 39.00)	0.988
12week	48.00 (27.00, 72.00)	38.00 (26.50, 64.75)	0.517
24week	40.00 (25.50, 59.50)	38.50 (22.00, 51.00)	0.338
36week	32.00 (22.25, 51.75)	25.00 (17.75, 42.50)	0.109
48week	32.50 (22.75, 45.75)	32.00 (20.00, 51.00)	0.658
60week	26.00 (20.00, 41.00)	26.00 (19.50, 46.00)	0.735
72week	26.00 (19.50, 43.00)	24.50 (16.75, 39.00)	0.323
84week	28.00 (19.00, 43.00)	22.00 (15.50, 34.75)	0.171
96week	23.00 (17.00, 30.00)	23.00 (18.00, 27.50)	0.913

### Association of hepatic steatosis with HBsAg clearance

**Figure 5:**
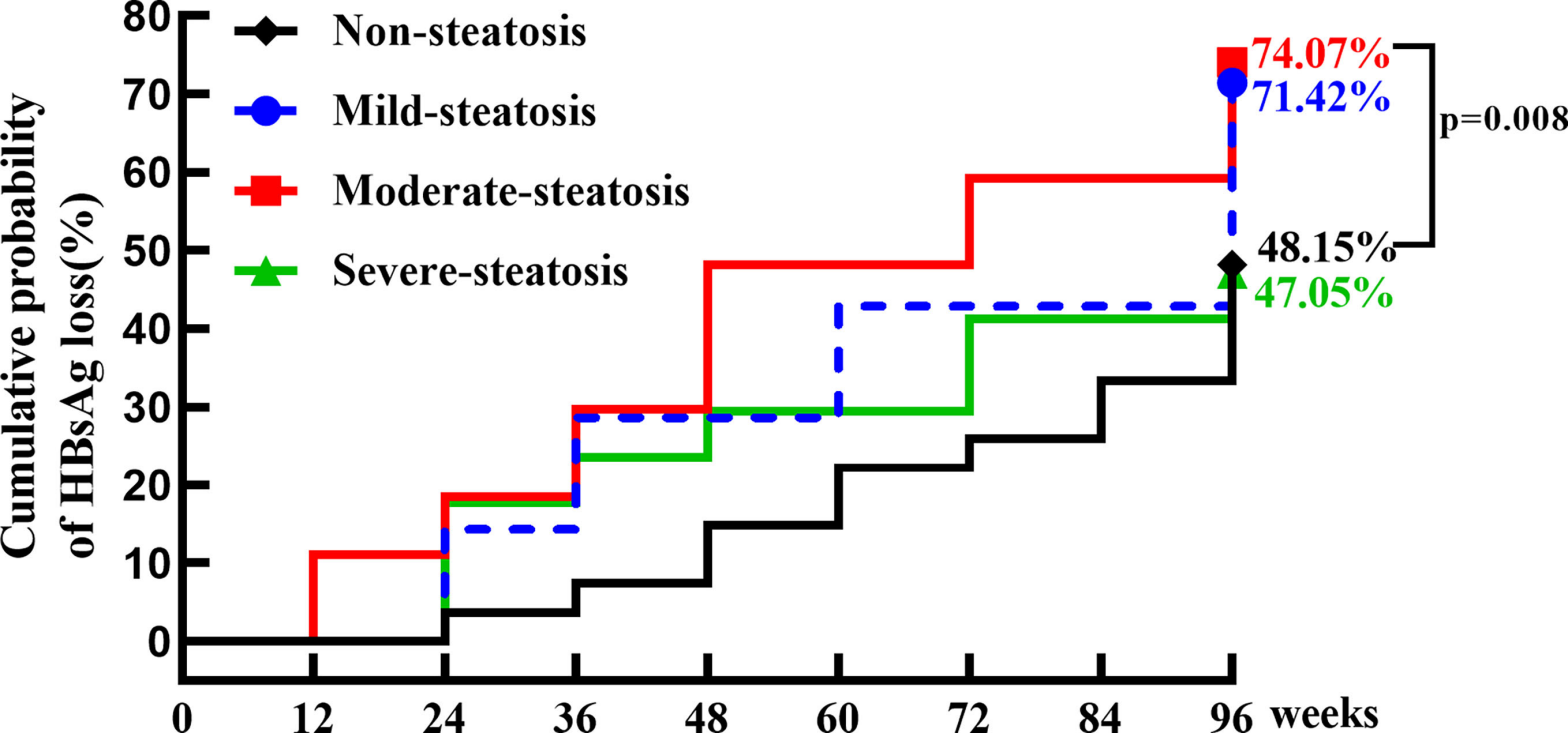
The cumulative incidence of HBsAg clearance in IHCs without and with hepatic steatosis (mild, moderate, severe). The cumulative incidence of HBsAg clearance was 48.15% (13/27), 71.42% (5/8), 74.07% (19/26) and 47.05% (8/17) in IHCs without and with mild, moderate, severe hepatic steatosis. The rate of HBsAg clearance was significantly higher in IHCs with moderate hepatic steatosis than in IHCs without hepatic steatosis (74.07% vs 48.15%, p = 0.008).

At baseline, 78 patients in treatment group were divided into four groups according to CAP value, 27 with no hepatic steatosis, 8 with mild steatosis, 26 with moderate steatosis and 17 with severe steatosis. The proportion of patients with moderate steatosis in R group (42.2%) was significantly higher than that in NR group (21.2%) (p=0.033). The cumulative clearance rate was 62.47% at week 96 in IHCs with hepatic steatosis (mild, moderate, and severe), compared to 48.15% of those without steatosis (p=0.058). The cumulative HBsAg clearance rate was 71.42%, 74.07% and 47.05% at week 96 in patients with mild, moderate, and severe steatosis, respectively. IHCs with moderate steatosis was most sensitive to PEG-IFN, the HBsAg clearance rate in moderate steatosis was significantly higher compared with that in no-steatosis group (P=0.008). (**Table 4**; **Figure 5**)

**Table 4:** The proportion of patients with different level of CAP in R and NR group at baseline.

CAP (dB/m)	R group n=45	NR group n=33	P-value
<238	28.89 (13.00/45.00)	42.42% (14.00/33.00)	0.214
238-250	11.11% (5.00/45.00)	9.09% (3.00/33.00)	0.771
251-292	42.22% (19.00/45.00)	21.21% (7.00/33.00)	0.033*
>292	17.78% (8.00/45.00)	27.27% (9.00/33.00)	0.316

* p <0.05.

### Safety

Six of 84 individuals who received PEG-IFN treatment stopped the medication because of side effects. Four patients suffered hyperthyroidism and one patient developed hypothyroidism. The level of TPOAb was increased by at least 10-fold in one patient. HBsAg clearance was obtained in two patients with hyperthyroid when PEG-IFN was discontinued. During the follow-up period, thyroid function and thyroid antibodies were gradually back to normal in six individuals with aberrant thyroid function (**Table 5**).

**Table 5:** Adverse effect of the study population.

Patients	Gender	Age	baseline HBsAg(IU/ml)	adverse effect	The time point of treatment discontinuation	EOT HBsAg(IU/ml)	Whether the adverse reaction is improved	96WHBsAg(IU/ml)
1	female	46	142.90	Hypothyroidism	Week24	132.7	YES	135.10
2	male	47	79.63	Hyperthyroidism	Week24	42.25	YES	28.60
3	male	60	23.62	Hyperthyroidism	Week48	<0.05	YES	<0.05
4	male	47	8.41	TPOAb raised	Week48	<0.05	YES	<0.05
5	female	31	45.78	Hyperthyroidism	Week60	0.079	YES	0.44
6	female	37	3.47	Hyperthyroidism	Week60	0.078	YES	0.06

## Discussion

PEG-IFN-treated IHCs, especially those with low level of baseline HBsAg, had higher HBsAg clearance rate. In our previous study, 134 IHCs with HBsAg <1000 IU/ml were treated with PEG-IFN alone or in combination with adefovir (ADV), and the clearance rate of HBsAg reached 44.7% at week 96 (10); Dang’s study (11) showed that IHCs with HBsAg < 1500IU/ml treated with PEG-IFN for 72 weeks, the HBsAg clearance and seroconversion rates were 37.4% and 29.7%. In addition, the clinical cure rate of PEG-IFN treatment in IHCs with lower HBsAg levels has also been studied. Zeng (12) achieved a high HBsAg clearance rate of 93.8% in IHCs with HBsAg < 20 IU/mL (n=32) treated with PEG-IFN for 48 weeks. But it should be pointed out that patients with HBsAg < 20 IU/ml are rare because of too low HBsAg in the clinic and the study sample size was only 32 cases. In order to explore the effect of PEG-IFN treatment in more clinical situation, this study explored IHCs with HBsAg < 200IU/ml. The cumulative clearance rate at week 96 was 57.69% (45/78), suggesting that IHCs with HBsAg < 200 IU/ml at baseline could achieve a high rate of HBsAg clearance with PEG-IFN treatment.

Previous studies have suggested that elevated ALT during PEG-IFN treatment may indicate a better curative effect. A multicenter retrospective study of 121 HBeAg-positive CHB patients treated with PEG-IFN in the United States found that patients with acute ALT elevation after PEG-IFN treatment had a sustained HBV DNA response and significantly improved HBeAg and HBsAg clearance (13). A global, multicenter, randomized, controlled study of HBeAg-positive patients treated with tenofovir disoproxil fumarate (TDF) plus PEG-IFN for 48 weeks, patients with acute ALT elevation within 24 weeks had higher HBeAg clearance rate (38.9% vs 10.4%) and HBsAg clearance rate (24.1% vs 1.7%) than those without acute ALT elevation (14). This study also found that IHCs treated with PEG-IFN showed a significant elevation of ALT at week 12 both in R and NR group (p < 0.001, p=0.001). Although there was no statistically significant difference of ALT level between the two groups at each time point, it was seen that the proportion of patients with elevated ALT was higher in R group (48.72%) than NR group (34.37%) at early treatment.

Most current studies suggest that hepatic steatosis is conducive to spontaneous clearance of HBsAg. A study by Chu et al. (15) showed that spontaneous HBsAg clearance occurred in 54 of 162 IHCs. IHCs with moderate (OR=3.22, P=0.017) and severe (OR=3.87, P=0.041) steatosis were found to be more than threefold of HBsAg clearance rate than those without hepatic steatosis, while mild steatosis was not significantly associated with HBsAg clearance (OR=1.76, P=0.157). Li et al. (16) in a retrospective cohort study (including 6,786 Asian CHB patients) found that 10-year cumulative HBsAg clearance was significantly higher in CHB patients with comorbidity of fatty liver than those without fatty liver (15.91% vs 11.84%, p=0.003). However, whether steatosis affects antiviral efficacy remains controversial. Nguyen et al. (17) showed that steatosis was a significant predictor for HBsAg clearance (HR=1.84, 95% CI 1.03-3.29) in CHB patients treated with entecavir (ETV)/TDF. In a Chinese study that enrolled 213 patients with CHB treated with ETV, HBV DNA clearance and HBeAg seroconversion rates were significantly lower in patients with comorbidity of steatosis than those without (18). A study by Lin et al. (19) demonstrated that the HBeAg clearance rate and HBV DNA inhibition rate did no differ in with or without steatosis treated with nucleos(t)ide analogue (NAs) (LAM/ADV/LdT/ETV/TDF) monotherapy. Previous studies on whether steatosis affects antiviral efficacy were mainly based on NAs treatment with HBV DNA suppression and HBeAg seroconversion as indicators. However, it has not been reported whether steatosis affects HBsAg clearance for IHCs with PEG-IFN therapy. In this study, the cumulative clearance rate of HBsAg at week 96 in patients with baseline steatosis (CAP>238 dB/m) had higher tendency than that in patients without steatosis. However, moderate steatosis definitively benefits the HBsAg clearance in patients treated with PEG-IFN.

Some adverse effects may occur during the treatment of PEG-IFN, and thyroid dysfunction is one of the common adverse reactions (20). After discontinuation of interferon therapy, thyroid function and antibodies returned to normal. Therefore, the adverse reactions should be closely monitored during the application of PEG-IFN, and the clinical cure of hepatitis B should be pursued safely and effectively.

In conclusion, PEG-IFN is effective on IHCs with HBsAg< 200 IU/ml. IHCs with moderate hepatic steatosis are more sensitive for the treatment.

## Data availability statement

The original contributions presented in the study are included in the article/supplementary material. Further inquiries can be directed to the corresponding authors.

## Ethics statement

The studies involving human participants were reviewed and approved by The research ethics committee of Beijing Youan Hospital, Capital Medical University, China ([2017]24). The patients/participants provided their written informed consent to participate in this study.

## Author contributions

ZC designed research. HL, LL and SL analyzed the results; YZ and DZ conducted the experiments. HL, LL and SL wrote the manuscript. YL guided statistical analysis. JZ, XC and ZC contributed to the interpretation of the results and critical revision of the manuscript for important intellectual content. All authors read and approved the final manuscript. All authors contributed to the article and approved the submitted version.
